# Bicycling crashes on streetcar (tram) or train tracks: mixed methods to identify prevention measures

**DOI:** 10.1186/s12889-016-3242-3

**Published:** 2016-07-22

**Authors:** Kay Teschke, Jessica Dennis, Conor C. O. Reynolds, Meghan Winters, M. Anne Harris

**Affiliations:** School of Population and Public Health, University of British Columbia, 2206 East Mall, Vancouver, BC V6T 1Z3 Canada; Dalla Lana School of Public Health, University of Toronto, Toronto, Canada; Institute for Resources, Environment and Sustainability, University of British Columbia, Vancouver, Canada; Faculty of Health Sciences, Simon Fraser University, Burnaby, Canada; School of Occupational and Public Health, Ryerson University, Toronto, Canada

**Keywords:** Bicycling injuries, Bike safety, Bike lanes, Public transport, Streetcar, Train, Built environment

## Abstract

**Background:**

Streetcar or train tracks in urban areas are difficult for bicyclists to negotiate and are a cause of crashes and injuries. This study used mixed methods to identify measures to prevent such crashes, by examining track-related crashes that resulted in injuries to cyclists, and obtaining information from the local transit agency and bike shops.

**Methods:**

We compared personal, trip, and route infrastructure characteristics of 87 crashes directly involving streetcar or train tracks to 189 crashes in other circumstances in Toronto, Canada. We complemented this with engineering information about the rail systems, interviews of personnel at seven bike shops about advice they provide to customers, and width measurements of tires on commonly sold bikes.

**Results:**

In our study, 32 % of injured cyclists had crashes that directly involved tracks. The vast majority resulted from the bike tire being caught in the rail flangeway (gap in the road surface alongside rails), often when cyclists made unplanned maneuvers to avoid a collision. Track crashes were more common on major city streets with parked cars and no bike infrastructure, with left turns at intersections, with hybrid, racing and city bikes, among less experienced and less frequent bicyclists, and among women. Commonly sold bikes typically had tire widths narrower than the smallest track flangeways. There were no track crashes in route sections where streetcars and trains had dedicated rights of way.

**Conclusions:**

Given our results, prevention efforts might be directed at individual knowledge, bicycle tires, or route design, but their potential for success is likely to differ. Although it may be possible to reach a broader audience with continued advice about how to avoid track crashes, the persistence and frequency of these crashes and their unpredictable circumstances indicates that other solutions are needed. Using tires wider than streetcar or train flangeways could prevent some crashes, though there are other considerations that lead many cyclists to have narrower tires. To prevent the majority of track-involved injuries, route design measures including dedicated rail rights of way, cycle tracks (physically separated bike lanes), and protected intersections would be the best strategy.

## Background

Both bicycling and public transport are seen as means to increase physical activity in the population and to reduce air emissions that induce climate change [1]. Among public transport modes, streetcars (North American term) or trams (European term) have many benefits (higher ridership and low carbon footprint) and are often promoted over buses [2, 3]. Bicycling is often identified as a means to access public transport, but the potential impact of a transit system on bicycling injuries has rarely been studied. In recent years, research has begun to report high numbers and high risks of injuries to bicyclists riding near streetcar or train tracks [3–9].

These studies include one we conducted in the cities of Vancouver and Toronto, Canada [4, 6, 7]. The study included 690 adults who were sufficiently injured in a bicycling crash to require treatment at a hospital emergency department. Of these, 14 % had a crash that directly involved streetcar or train tracks [6]. A three-fold increased risk of injury was observed when cycling on routes with streetcar or train tracks, with a higher risk in the street segments between intersections than at intersections themselves [4, 7]. Unlike Vancouver, Toronto has an extensive streetcar system (the largest in North America) and 32 % of participating cyclists injured there had crashes that directly involved tracks, compared to 2.5 % in Vancouver.

In this paper, we examine the Toronto crashes in more detail, to describe the circumstances, infrastructure at the crash sites, trip conditions, and characteristics of the injured cyclists and their bicycles. Our goal was to identify factors that are associated with increased or decreased potential for crashes on tracks. We also obtained engineering information about the tracks from the transit authority and visited a sample of bicycle shops to measure tire sizes and interview staff about this issue. The overall aim was to identify ways to prevent such crashes in the future.

## Methods

### The injury study

The procedures for the injury study have been described in detail elsewhere [4, 6, 7]. The following is a summary of the Toronto component relevant to the analyses presented in this paper. The study population consisted of adult (≥19 years) residents of Toronto who were injured while riding a bicycle in the city and treated within 24 h in the emergency departments of St. Michael’s Hospital, Toronto General Hospital or Toronto Western Hospital between May 18, 2008 and November 30, 2009. All hospitals were located in the central business district, and one was a regional trauma centre.

Eligible participants were interviewed in person by trained interviewers about personal characteristics, trip conditions, and crash circumstances, using a structured questionnaire [10] as soon as possible after the injury to maximize recall. Crash circumstances were derived from participants’ answers to the following questions:

- In your own words, please describe the circumstances of the injury incident.
- Was this a collision between you and a motor vehicle, person, animal or object (including holes in the road)?
- If yes, what did you collide with? (response options: car, SUV, pick-up truck, or van; motorcycle or scooter; large truck; bus or streetcar; pedestrian; cyclist; animal; other non-motorized wheeled transport; pot hole or other hole; streetcar or train track)

Personal characteristics queried in the interview and used in analyses presented here included sex, age, and three factors potentially related to cycling skill: whether they had taken an urban cycling training course; whether they considered themselves an experienced cyclist; and the number of times they had cycled in the last year (reported by season and summed). Trip characteristics included weather, purpose, bike type, and whether medications, alcohol or marijuana were used in the 6 h prior. Bike type was queried with a poster of photos showing an example of each of the following types: city, touring, hybrid, racing, folding, cruiser, mountain and recumbent. Participants were also able to specify other bike types.

Structured site observations were made to document characteristics of injury and control sites, and allow route infrastructure classification [4, 7, 11]. The observations were made blind to whether an injury took place at the site or not. In the current analyses, only the injury site data were used. Infrastructure characteristics used in these analyses were selected primarily if they were shown to be related to injury risk in previous analyses: route type, intersection location or not, grade, and presence of construction [4, 7]. Route type at an intersection was defined as the route type the cyclist arrived from.

To determine whether a crash directly involved streetcar or train tracks, several steps were involved [6]. The blinded and objective site observation data were used to identify whether the crash was at a site where tracks were present. Wherever this was the case, the closed-ended interview response about what the participant collided with and the open-ended interview response describing the crash circumstances were reviewed. Each crash resulting from the participant’s bike slipping on a track rail, hitting a track component, or its tire being caught in the rail flangeway was separately coded and counted. These circumstances were classified as “injury directly involved a streetcar or train track”; all other injuries were classified as “other or unknown injury circumstance”. The interview data were also used to classify whether a motor vehicle was involved in the crash (i.e., a collision with a motor vehicle or a crash after a maneuver to avoid a collision with a motor vehicle).

Data analyses were performed using JMP 11 (SAS Institute, Cary, NC). Descriptive data on crash circumstances, infrastructure at the crash site, trip conditions and personal characteristics were compared for injuries involving streetcar or train tracks *vs.* other or unknown circumstances. The Chi^2^ test (categorical independent variables) and *t*-test (continuous independent variables) were used to identify factors that differed between categories of the dependent variable: injury directly involved streetcar or train tracks *vs.* other or unknown injury circumstances. Variables that were significant in the bivariate analyses (*p* < 0.05) were offered to multiple logistic regression. Two independent variables were strongly associated with each other (experienced cyclist and cycling frequency). Of the two, only cycling frequency was significant in multiple regression and retained in the final model.

### Streetcar tracks in Toronto

The Toronto Transit Commission Streetcar Department was contacted to obtain the engineering specifications of the streetcar rails and flangeways (gap in the road surface alongside rails) and other characteristics of the streetcar rail infrastructure, and to provide comparisons to train infrastructure in the city.

### Survey of Toronto bike shops

In the summer of 2015, we sought input from eight bicycle shops within 7 km of the Toronto central business district and recognized by investigators as frequented by commuter cyclists. Five of the shops were in the downtown core, two to the east, and one to the north. Each shop was sent an introductory letter explaining the purpose of the survey and the procedures involved. This was followed with a phone call requesting participation and setting up a time to visit the shop. The survey was open to all shop employees; the position of the staff member interviewed was not recorded. The following open-ended questions were asked:

- What types of cyclists shop at this store?
- Do any shoppers ask about ways to avoid streetcar track injuries? What advice do you give? To your knowledge, does this store have a policy or standard recommendation for customers concerned about streetcar track injuries?
- There are hundreds of tire sizes and styles. Do you sell any tires (or do you know of any tires) that are less likely to get caught in a streetcar track groove or slip on streetcar track surfaces?
- Do you think there are any other bicycle, wheel, or tire design elements that could reduce the risk of streetcar track injuries?

We asked to be shown popular bikes and to measure their tires. The following data were recorded: bike type; tire manufacturer; tire size as imprinted on the tire; and measured tire width (including knobs, if present). Bike type was recorded as described by bike shop personnel. Measurements (tire width, tire depth, wheel width) were taken on inflated tires mounted on bicycle wheels, using calipers (Capri Tools, CP20001, Pomona, California). Tire widths were summarized overall and by bike type as means, minima and maxima. Overall proportions narrower and wider than Toronto streetcar flangeways were calculated. The measured tire widths were compared to the widths printed by the manufacturer on the tire.

## Results

### Streetcar and train tracks in Toronto

**Fig. 1:**
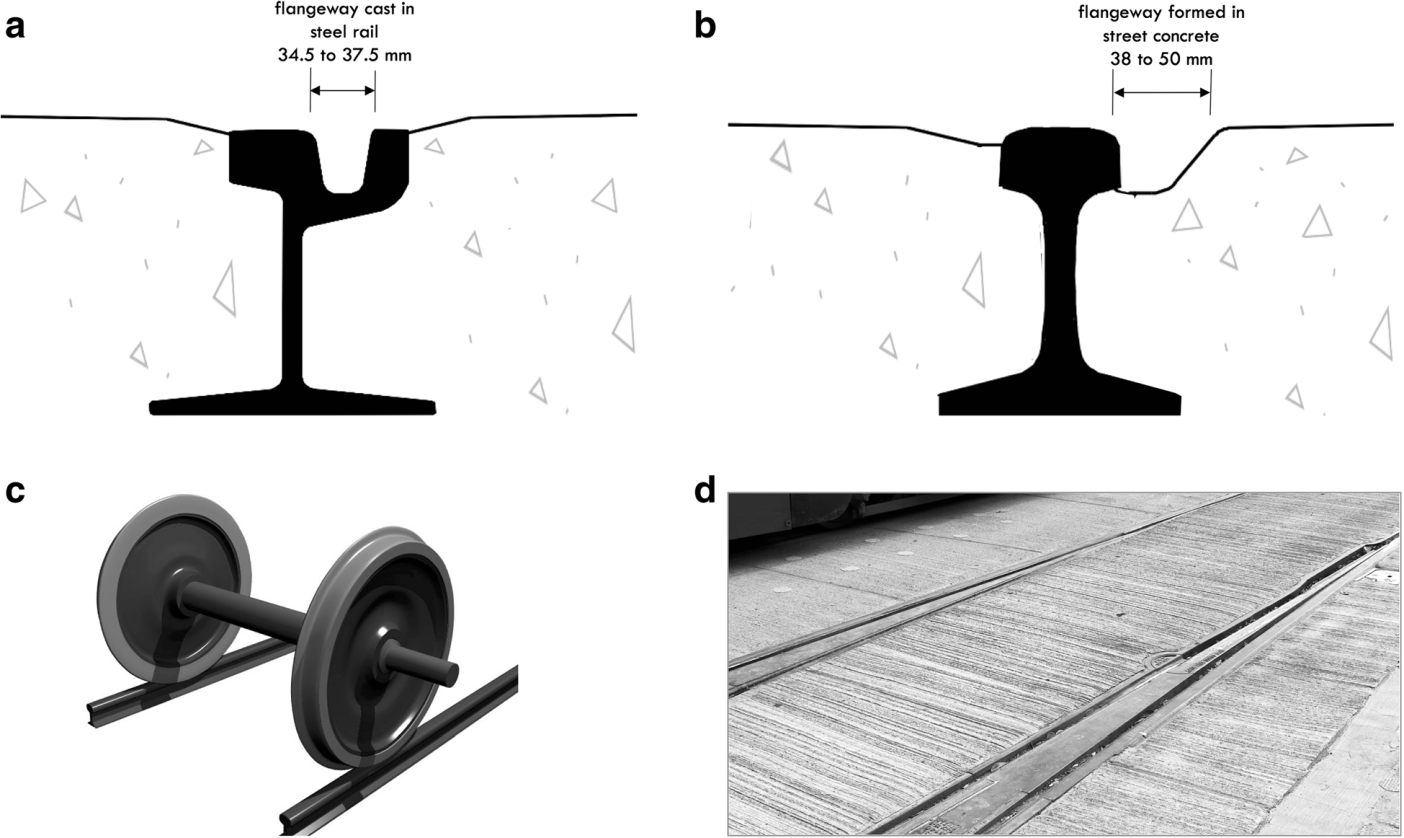
Toronto rails, flangeways and wheels. **a** Profile of girder rail (NP4aMOD) with integrated flangeway. **b** Profile of tee rail (115 lb AREA) showing flangeway created by gap between side of rail and adjacent concrete. **c** Streetcar or train wheels on tee rail, showing larger diameter flanges that hold wheels in place. **d** Example of how flangeway widths can vary along a streetcar line. (Image c: Wikimedia Commons, Pantoine)

Toronto’s streetcar system has about 80 km of double track (one set in each direction) (personal communication, Stephen Lam, Toronto Transit Commission, May 2015). Most of the eleven routes operate in mixed traffic, but three operate in dedicated rights of way (for streetcars only, except at intersections). Streetcar tracks in Toronto are typically constructed with two types of rail (Fig. 1): girder rails and tee rails. Wheels ride on the rail surface and are held in position by a larger diameter flange on one side of the wheel (Fig. 1c) that rides in a slot beside the rail: the “flangeway”. In girder rails, the flangeway is part of the cast steel rail, whereas flangeways for tee rails are constructed as they are laid in the street concrete. Most straight sections of Toronto streetcar tracks are constructed with tee rails and most curves use girder rails. The flangeway of girder rail used in Toronto has a typical width at the top of 37.5 mm (millimeter) and a width just below the surface of 34.5 mm, though the width changes as the railhead is worn. Tee rail flangeways vary in width; US guidelines indicate widths in similar systems of 38 to 50 mm [12].

Railway train rights of way are private except at road crossings (personal communication, Stephen Lam, Toronto Transit Commission, May 2015). Tracks are constructed with tee rails that sit atop railway ties for most of their distance. At road crossings, the rail is embedded in the road surface material (concrete or asphalt) and flangeways are constructed as for streetcar tracks. Railways in Canada can use the same tee rail as the Toronto streetcar system (Fig. 1b), though on high tonnage lines, heavier rail with the same profile is used [13].

### The injury study

Our study included 276 people who had been injured while cycling and attended one of the three participating Toronto emergency departments. Of these, 139 had crashes at sites where streetcar or train tracks were present and 87 of those had crashes that directly involved the tracks. Three of the people who crashed at a streetcar track location could not remember enough detail about their crash to determine if the tracks were directly involved; these were classified as having unknown circumstances. None of the study participants had a collision with a streetcar or train.

The vast majority (85 %) of the track crashes resulted from the bicycle tire being caught in the flangeway (Table 1). The remainder resulted from tires slipping on the rail surface (just over half of slips occurred during rain, snow or fog conditions). Table 1 provides sample descriptions of the circumstances leading to track-involved crashes. None of the track crashes included collisions with other parties, but a common feature was sudden maneuvers to avoid collisions (mainly with motor vehicles, but also cyclists and pedestrians); these resulted in unanticipated track crossings or crossings at shallower angles than planned.

**Table 1:** Circumstances of crashes directly involving streetcar (*N* = 83) or train tracks (*N* = 4), with examples^a^

Tire caught in track flangeway, *N* = 74 (85.1 % of track injuries)
Intersection examples
I had been cycling on the right side of the road but I wanted to make a left turn and while moving to the centre of the lane my bike wheels got caught in the streetcar tracks.
As I approached an intersection, there was a car in front of me turning right. To go straight, I moved around the car into the left lane but as I did, my front tire got stuck in the streetcar track.
I had a green light so I proceeded through the light. A cyclist turned right onto the bike path I came from. I swerved to avoid her and my wheel got caught in the train track.
Non-intersection examples
As I was cycling in the curb lane, a truck passed me, stopped and turned on his hazards. I went around him on the left which put me between the street car tracks. As I was going over the street car tracks my back wheel got caught.
There was an ambulance coming from behind me and a car parallel parking in front of me. I moved across the tracks to avoid the car. When I attempted to move back into the right lane, my back wheel got caught in the streetcar track.
I was biking in the right hand lane and in front of me a woman opened her car door. I moved to the center lane, but as I was moving back to the right lane my front tire got caught in a streetcar track.
There were three big trucks parked in the curb lane. I moved into the lane beside me to avoid them. I looked back to move back into the curb lane when my front tire got caught in the streetcar tracks.
There was another cyclist ahead of me. I moved into the left lane and passed her. When I attempted to move back into the right lane, my front wheel got caught in a street car track.
Tire slipped on track rail, *N* = 13 (14.9 % of track injuries)
The roads were very wet and slick. I was travelling south, turning left. I was leaning into the turn. I hopped over the first streetcar rail and was getting ready to cross the next rail when my back tire slipped on the track.
I came to two sets of railway tracks. I slowed down and crossed the first set but when I started to cross the second set, my front tire slid on the wet track.

^a^As described by injured cyclists in Toronto

**Fig. 2:**
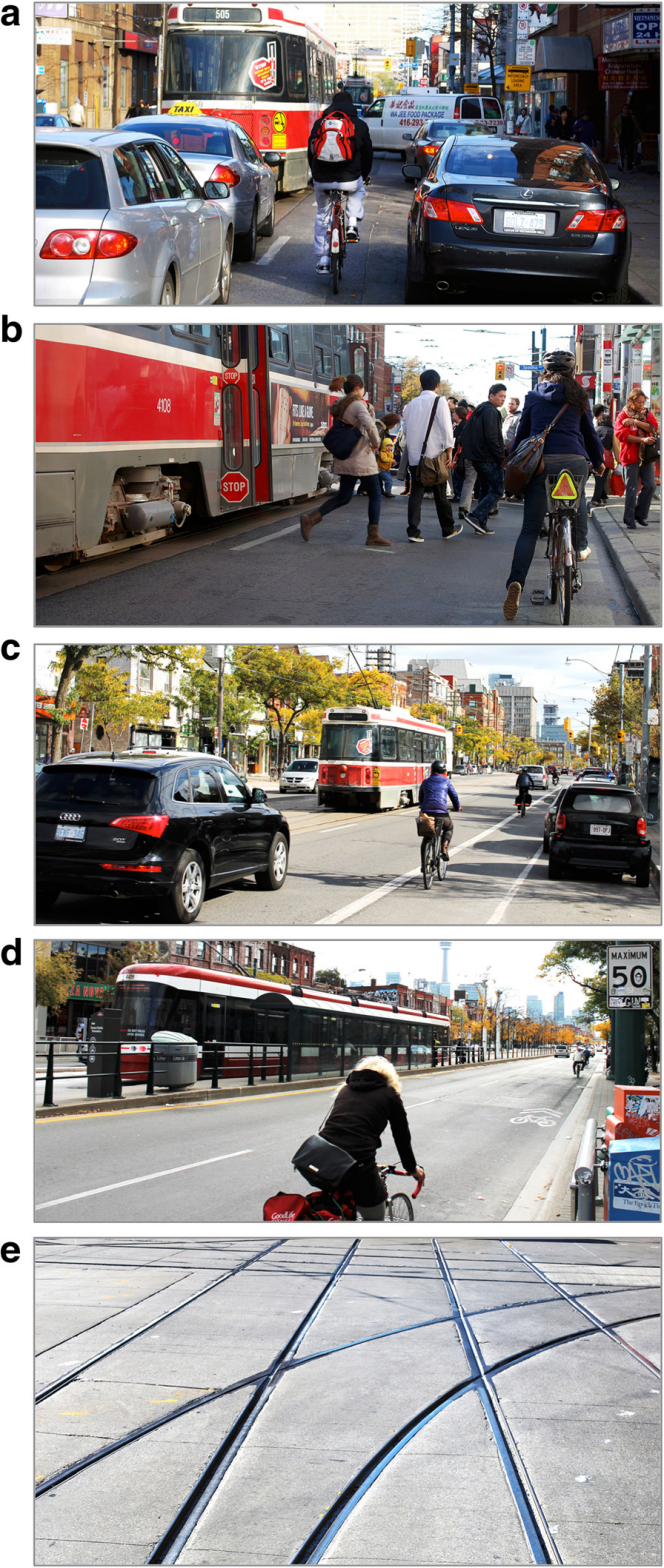
Examples of route types with streetcars in Toronto. **a** Major street with parked cars, no bike infrastructure. **b** Major street, no parked cars, no bike infrastructure. **c** Major street with painted bike lane. **d** Major street with dedicated streetcar right of way. **e** Complex network of rails and flangeways at intersection of two streets with streetcar lines. (Photos a, b, e: Wikimedia Commons, Hallgrimsson)

Table 2 summarizes data on the crash circumstances, route infrastructure at the crash site, trip conditions, and personal characteristics of the cyclists injured in streetcar or train track crashes *vs.* in other circumstances. Six factors had significant associations with crashes that directly involved tracks. A higher proportion of track crashes than other crashes were on major streets with parked cars and no bike infrastructure (Fig. 2). A slightly higher proportion of track crashes than other crashes were at intersections, mainly because of a much higher proportion of left turn crashes. Bike types with higher proportions involved in track crashes were hybrid, racing, and city bikes. Women and inexperienced cyclists had higher proportions of track crashes. People who had track crashes cycled on average less frequently than those who had other crash circumstances.

**Table 2:** Crash circumstances, crash site infrastructure, trip conditions and cyclist characteristics, 276 cyclists injured in Toronto

	Crash directly involved streetcar or train track		Other or unknown crash circumstance	
	N	%	N	%
	87	^a^31.5	189	^a^68.5
Crash circumstances				
Motor vehicle involved	36	^b^41.4	97	^c^51.9
Infrastructure at Crash Site				
Route type^d^				
Major street with parked cars, no bike infrastructure	49	56.3	53	28.0
Major street, no parked cars, no bike infrastructure	26	29.9	58	30.7
Major street with painted bike lane	7	8.0	29	15.3
Residential street	2	2.3	20	10.6
Sidewalk or multiuse path	3	3.4	29	15.3
Intersection status^d^				
Non-intersection	59	67.8	143	75.7
Intersection, straight through	13	14.9	42	22.2
Intersection, right turn	2	2.3	3	1.6
Intersection, left turn	13	14.9	1	0.5
Grade				
Downhill	28	32.2	75	39.7
Flat	52	59.8	91	48.1
Uphill	7	8.0	23	12.2
Construction present	13	14.9	21	11.1
Trip Conditions				
Weather during trip				
Clear	52	59.8	127	69.4
Cloud cover	20	23.0	35	19.1
Rain, snow or fog	12	13.8	13	7.1
Wind	3	3.4	8	4.4
Trip purpose				
Multiple	4	4.6	12	6.3
Personal business	21	24.1	31	16.4
Recreation	9	10.3	33	17.5
Social reasons	11	12.6	37	19.6
Commute to work or school, job	42	48.3	76	40.2
Bike type used on trip^d^				
Hybrid	37	42.5	49	25.9
Racing, track, fixed gear	14	16.1	24	12.7
City	6	6.9	9	4.8
Mountain	17	19.5	57	30.2
Touring/road	9	10.3	32	16.9
Other: BMX, cruiser, folding	4	4.6	18	9.5
Drugs or alcohol used in 6 h prior to trip				
Medication	5	5.7	17	9.0
Alcohol	10	11.5	18	9.5
Marijuana	1	1.1	4	2.1
Cyclist characteristics				
Age				
20–29	32	36.8	60	31.7
30–39	26	29.9	57	30.2
40–49	13	14.9	29	15.3
50–59	11	12.6	26	13.8
60 +	5	5.7	17	9.0
Sex^d^				
Female	52	59.8	69	36.7
Male	35	40.2	119	63.3
Urban cycling training course taken				
Yes	5	5.7	7	3.7
No	82	94.3	182	96.3
Experienced cyclist^d^				
Yes	74	85.1	181	95.7
No	13	14.9	8	4.2
Cycling frequency (times per year)^d^	mean 123 (SD 72.4)		mean 149 (SD 74.0)	

^a^ % of all 276 injured cyclists

^b ^% of 87 injuries on tracks (applies to all following % in this column)

^c ^% of 189 injuries not on tracks (applies to all following % in this column)

^d ^Variable distribution significantly different (*p* < 0.05) for “crash directly involved streetcar or train track” *vs.* “other or unknown crash circumstance”

Other characteristics did not significantly differ between track-involved and other crash circumstances, but provide a picture of the crashes. Of track crashes, 41 % involved motor vehicles, 60 % occurred on flat grades, and 15 % occurred where construction was present. Most track crashes happened on clear days (60 %), and about half happened on commutes to work or school or on the job. Cyclists had rarely used prescription medications (6 %), alcohol (12 %) or marijuana (1 %) in the 6 h prior to the trip. The majority of those injured on streetcar or train tracks were ages 20 to 39 (67 %). Few had urban cycling training (6 %).

In multiple logistic regression (Table 3), route type, intersection status, sex and cycling frequency were significantly associated with whether a crash directly involved a streetcar or train track vs. not. Major streets with parked cars and no bike infrastructure had higher odds of a track-involved crash than all other route types. Left turns at intersections had much higher odds of a track-involved crash compared to other intersection movements and non-intersections. People who cycled more frequently had lower odds of a track-involved crash. Women had higher odds than men of a track-involved crash.

**Table 3:** Factors associated with crashes directly involving streetcar or train track vs. other or unknown circumstances

	OR^a^	95 % CI^b^
Route type		
Major street with parked cars, no bike infrastructure	1.0	ref
Major street, no parked cars, no bike infrastructure	**0.44**	**(0.22, 0.86)**
Major street with painted bike lane	**0.15**	**(0.04, 0.43)**
Residential street	**0.12**	**(0.02, 0.46)**
Sidewalk or multiuse path	**0.12**	**(0.03, 0.38)**
Intersection status		
Non-intersection	1.0	ref
Intersection, straight through	0.84	(0.38, 1.77)
Intersection, right turn	1.03	(0.12, 7.26)
Intersection, left turn	**43.4**	**(7.54, 838)**
Sex		
Male	1.0	ref
Female	**2.10**	**(1.13, 3.92)**
Cycling frequency		
Additional 100 times cycling per year	**0.67**	**(0.44, 0.99)**

^a ^Odds ratios (OR) from multiple logistic regression, *N* = 276 injured cyclists in Toronto. Bold indicates odds ratio is statistically significant

^b ^95 % confidence interval (CI)

### Survey of Toronto bike shops

Seven of the eight invited bike shops participated in the survey. As expected given the shop selection, commuter cyclists were their main customers (50 to 85 %) and they mainly bought commuter, hybrid, comfort or city bikes. Recreational cyclists were the next largest group (15 to 50 %), buying mountain bikes in addition to the bike types typically purchased by commuters. Smaller niche groups bought fat, road, racing, or recumbent bikes or trikes. Most stores had more male than female customers, though several reported 40 % or more of their customers as women. One store catered to families.

All bike shop personnel reported that some customers, particularly those who were less experienced, asked about how to handle streetcar tracks, including asking whether wider tires or mountain bikes would help. All indicated that they advised such customers to be aware of tracks and cross them at appropriate angles: at least 45°, 90° optimally. Shop personnel reported selling wide tires to concerned customers, but many were reluctant to advise this, for several reasons: they considered wide tires to be slower and less efficient for commuting; they thought very wide tires (e.g., fat bike tires that are over 100 mm wide) would be needed to avoid being caught in all flangeways; they thought that some wide tires may still get caught especially if they have knobs on them; and they thought that wide tires would still have the risk of slipping on track surfaces. Other advice that shop personnel reported giving to concerned customers included planning their routes to avoid streets with streetcar tracks, making two-stage left turns at intersections with tracks, being extra cautious in wet and icy weather, and keeping tires inflated. Most shop personnel indicated that there were no tires that could prevent slipping on rail surfaces, but one mentioned slick tires with grooves to help move water away from the center, and two mentioned tires made from “tackier” rubber compounds, though they thought these wear more quickly.

Table 4 summarizes width measurements of tires on bikes commonly sold at the participating shops. The widths spanned a wide range, from 24.8 mm on a single speed racing style bike to 112 mm on a fat bike. Most of the tires were narrow enough to be caught in the narrowest streetcar track flangeways (34.5 mm) and only a few were wider than the widest likely flangeways in Toronto (50 mm). Some bike types had consistent tire sizes (e.g., single speed bikes had consistently narrow tires < 30 mm; touring or road bikes medium width tires from 33 to 37 mm; and cruiser bikes wide tires ≥ 49 mm), whereas others, particularly city and hybrid bikes, had broad ranges of tire widths. We compared measured tires widths with the manufacturer widths imprinted on the tire and found they agreed very well (mean |difference| = 1.7 mm, SD (standard deviation) = 1.1 mm, range 0 to 4.4 mm). Toronto has a public bike share program in the downtown core (1000 bikes in 2014); its bikes have 49.5 mm tires (personal communication, Scott Hancock, Motivate Company Toronto, January 2016).

**Table 4:** Measured tire widths of bicycles commonly sold in seven Toronto bike shops, by bike type

	N	Mean width (mm)	Minimum width (mm)	Maximum width (mm)	Proportion <34.5 mm^a^	Proportion > 50 mm^b^
All bike types	37	37.5	24.8	112	54 %	8 %
Single speed, fixed gear	5	27.1	24.8	29.8		
Commuter	9	34.4	29.9	38.8		
City	7	34.4	27.1	47.8		
Hybrid	8	35.2	25.6	55.7		
Touring, road	3	35.6	32.7	37.1		
Cruiser	2	54.5	49.0	60.0		
Other: comfort, cargo, fat	3	67.9	41.8	112		
Bike Share Toronto bikes^c^		49.5				

*mm* millimeter

^a ^Narrow enough to be caught in most Toronto streetcar flangeways

^b ^Widest flangeway specified in US guidelines for streetcar systems similar to Toronto’s [12]

^c ^Personal communication, Scott Hancock, Motivate Company Toronto, January 2016

## Discussion

The 87 cyclists injured in track-involved crashes in this study, recruited at three Toronto emergency departments over an 18-month period, appear to comprise the largest case series involving streetcar and train tracks reported to date. Three other studies identified cases in a single hospital over a similar time period: 41 emergency department cases in Sheffield [3]; ten hospitalized cases in Amsterdam [9]; and five emergency department cases (all e-bike users) in Bern [5]. A Dutch study in 13 hospitals reported on four emergency department cases [14]. As in our study, the dominant scenario for track crashes in most European studies was bike tires being caught in the flangeway [3, 5, 9]. The exception was that all four Dutch cases involved bicycle wheels being deflected by tram rails [14]. Two European studies described the types and severity of injuries (dominantly fractures and about a quarter of cases admitted to hospital) [3, 9]. We did not gather data on the types of injury, but we examined some aspects of injury severity in an earlier analysis [15]. We did not find greater severity (e.g., transport by ambulance, hospital admission) among cyclists injured at sites with streetcar or train tracks.

### Personal characteristics

We examined personal, trip and infrastructure characteristics related to track-involved crashes vs. other crash circumstances. Females were over-represented in track crashes (60 % female) compared to other crashes (37 %) and compared to the Toronto cycling population (34 %) [16]. The European studies did not provide comparisons, but reported that the majority of those injured on tracks were male [3, 5, 9]. We found that younger adults (ages 20 to 39, 67 %) were also somewhat over-represented in the track-involved crashes but not significantly so compared to other crashes (62 %). The injured cyclists in our study appear to have been younger than Toronto cyclists in general, based on a report that used different age categories (ages 15 to 34, 37 %), perhaps because our study area included large universities, many of whose students commute by bike [16].

Inexperience and less frequent cycling were associated with track-involved crashes in our study. The Sheffield study began immediately after their first tram line became operational and they found that cycling injuries peaked 3 to 6 months later, then declined by about 50 % [3]. They attributed this to media attention to the issue, supporting the idea that knowledge related to tracks could be helpful. Bike shop personnel contacted in this study all felt that the best protection for cyclists was to know how to behave near tracks, including being alert and crossing the tracks at a perpendicular angle. Similar guidance is provided in an Ontario government cycling skills guide, and adds waiting for breaks in traffic and potentially dismounting to cross tracks [17]. Since left turns can make perpendicular track crossing difficult (especially where there are complicated track patterns, Fig. 2e) and resulted in much higher odds of track-involved crashes, education materials could also encourage two-stage left turns.

Our results provide some support for the idea that increased knowledge or maneuvering skill may help, given that certain demographic groups were over-represented in track-involved crashes (e.g., less frequent cyclists, women), however a number of factors suggest education may not make a great difference. Those crashing on tracks were not especially inexperienced (average cycling frequency of 123 trips per year). Many of the crashes resulted from sudden maneuvers to avoid collisions with motor vehicles, other cyclists and pedestrians, situations that did not allow prior knowledge to be used as planned. Some cyclists (children, people with certain disabilities or who do not speak English) may not be reached by or be able to implement guidance about tracks. Finally, information about how to ride near tracks is long-standing and common in Toronto, yet the injury toll is very high. These caveats underscore the need for other approaches.

### Bike tire characteristics

Bike shop personnel reported that some cyclists request tires wide enough not to be caught in track flangeways. Our analyses showed that bike type was associated with whether injury circumstances were track-involved or not. Bike types more frequently in track-involved crashes had either consistently narrow tire widths (racing, single speed) or wide ranges of tire widths (hybrid, city) in the bike shop survey. Over half the tires on commonly sold bicycles were so narrow that they would fit in any of the track flangeways in Toronto. Although bike shop staff thought that only fat bike tires would be guaranteed not to be caught in the flangeways, tires of ~ 50 mm or greater on cruiser, comfort, and bike share bikes may reduce the likelihood of being caught in many, perhaps most, flangeways and are worthy of further study.

### Route characteristics

Route type was associated with track-involved crashes. On major streets with no bike infrastructure, it mattered whether there were parked cars or not (Fig. 2). These two route types have similar presence of streetcar or train tracks [7], but those without parked cars had less than half the odds of track-involved crashes. The absence of car parking provides cyclists with more room to maneuver and avoid track crashes when something unexpected takes place in front of them (Table 1). Removing car parking on streets with streetcar lines would improve conditions for cycling, especially if the space freed up were used for cycle tracks (as discussed below). Painted bike lanes, residential streets, and sidewalks or multiuse paths all had considerably lower odds of track-involved crashes than major streets with no bike infrastructure. This was almost certainly because these route types were much less likely to have streetcar or train tracks [7].

In the Netherlands, cyclists on major streets are typically provided cycle tracks (also called physically protected, segregated or separated bike lanes) and on-street tram lines typically have their own rights of way [14, 18]. This may account for the low numbers of tram-related crashes observed in the Dutch study [14]. Our injury study showed that cycle tracks greatly reduced injury risk to bicyclists [4, 7], but at the time of the study all examples of this infrastructure were in Vancouver, not Toronto. One Toronto streetcar line had its own right of way during the study period (Fig. 2) and all train lines did. We did a *post-hoc* check of whether any of the track-involved crashes were along these lines. None of track crashes between intersections were along them, but some were at their intersections (where there is no separation). Even if cycle tracks or designated rail rights of way would prevent only crashes that are not at intersections, track-involved crashes would be substantially reduced, since most (68 %) were not at intersections.

Left turns at intersections were highly overrepresented in track-involved crashes. This problem could also be addressed with Dutch-style infrastructure, often called “protected intersections”. Such intersections commonly feature corner islands that direct cyclists coming from cycle tracks to make two-stage left turns, as pedestrians do [19]. This would make it much easier to cross tracks at right angles, but could add long delays at intersections unless signal timing is optimized for cyclists and pedestrians, as in the Netherlands.

Protected intersections, cycle tracks and designated rail rights of way all follow the Swedish “Vision Zero” transport safety principle: acknowledging the inevitability of human error and providing route designs that minimize its consequences [20]. This vision aims to eliminate deaths and serious injuries related to transportation and is beginning to be adopted by other jurisdictions in Europe and North America.

### Strengths and limitations

This study benefitted from a large case series of track-involved crashes, a comparison group with other crash circumstances, and systematic data on crash circumstances, personal and trip characteristics, and route infrastructure at the crash sites. The mixed methods approach also collected data about advice provided to cyclists by bike shop personnel, widths of commonly sold bike tires, and engineering specifications of system rails and flangeways to provide a broader understanding of the problem and potential solutions.

Additional data would be helpful in future studies. We did not request data from the injured cyclists about their tire widths. This would be worthwhile to collect, so widths of tires involved in crashes can be compared to flangeway widths and risk related to tire width can be directly determined. Other characteristics (tire pressure, presence of tire knobs, weight of the cyclist) may alter the effective tire width and should be measured to see if they change the tire size needed to avoid being caught in flangeways. Direct measurements of flangeway widths throughout the rail system would be useful, though taking measurements in situ would be a dangerous endeavor. Similarly, field tests of different tire widths with bicycling track interaction maneuvers would be informative but risky to participants. In cities with streetcar or tram systems, it would be interesting to survey cyclists to see if they know how to reduce their individual risk of track crashes, and to survey planners and engineers to see whether they are familiar with design measures to reduce population risk of track crashes.

This study was conducted in one city in North America. Research conducted in other areas of the world with different cycling infrastructure, streetcar or tram infrastructure, and bicycle types would help determine whether these influence risk. Comparisons between cities and countries would be a great way to discover best practices. Unfortunately, such comparisons are difficult because the most common coding system for traffic injuries, the World Health Organization’s International Classification of Diseases [21], provides coding for collisions with a streetcar or train, but codes collisions involving tracks in a broad category of unspecified “stationary objects”. Crash and injury reporting systems that provide sufficient specificity to identify track-related crashes would allow administrative data to be used to tally these events, a crucial first step in understanding their impact [14].

## Conclusions

In a city with an extensive streetcar system, one-third of bicycling crashes directly involved streetcar or train tracks. Certain demographics were more likely to have track-involved crashes, suggesting that increased knowledge about how to avoid them might be helpful. However, such advice is long-standing and common in Toronto, yet the injury toll is very high, underscoring the need for other solutions. Tires wider than streetcar or train flangeways (~50 mm in the Toronto system) are another individual-based approach, but population-based measures are likely to provide the optimal solution. Our results showed that route infrastructure makes a difference to the odds of track-involved injuries. Dedicated rail rights of way, cycle tracks, and protected intersections that direct two-stage left turns are policy measures concordant with a Vision Zero standard. They would prevent most of the track-involved injury scenarios observed in this study.

## Abbreviations

CI, confidence interval; mm, millimeter; N, number; OR, odds ratio; SD, standard deviation

## References

[1] Maibach E, Steg L, Anable J (2009). Promoting physical activity and reducing climate change: Opportunities to replace short car trips with active transportation. Prev Med.

[2] Litman T. Rail transit in America: a comprehensive evaluation of benefits. Victoria Transport Policy Institute; 2015. http://www.vtpi.org/railben.pdf. Accessed 1 Feb 2016.

[3] Cameron IC, Harris NJ, Kehoe NJS (2001). Tram-involved injuries in Sheffield. Inj.

[4] Harris MA, Reynolds CCO, Winters M, Cripton PA, Shen H, Chipman M, Cusimano MD, Babul S, Brubacher JR, Friedman SM, Hunte G, Monro M, Vernich L, Teschke K (2013). Comparing the effects of infrastructure on bicycling injury at intersections and non-intersections using a case-crossover design. Inj Prev.

[5] Papoutsi S, Martinolli L, Braun CT, Exadaktylos AK (2014). E-bike injuries: Experience from an urban emergency department—A retrospective study from Switzerland. Emerg Med Int.

[6] Teschke K, Frendo T, Shen H, Harris MA, Reynolds CCO, Cripton PA, Brubacher JR, Cusimano MD, Friedman SM, Hunte G, Monro M, Vernich L, Babul S, Chipman M, Winters M (2014). Bicycling crash circumstances vary by route type: a cross-sectional analysis. BMC Public Health.

[7] Teschke K, Harris MA, Reynolds CCO, Winters M, Babul S, Chipman M, Cusimano MD, Brubacher J, Friedman SM, Hunte G, Monro M, Shen H, Vernich L, Cripton PA (2012). Route infrastructure and the risk of injuries to bicyclists: A case-crossover study. Am J Public Health.

[8] Vandenbulcke G, Thomas I, Int PL (2014). Predicting cycling accident risk in Brussels: a spatial case–control approach. Accid Anal Prev.

[9] Deunk J, Harmsen AM, Schonhuth CP, Bloemers FW (2014). Injuries due to Wedging of Bicycle Wheels in on-Road Tram Tracks. Arch Trauma Res.

[10] Bicyclists’ Injuries and the Cycling Environment Study, Cycling in Cities Research Program (2008). Interview Form.

[11] Bicyclists’ Injuries and the Cycling Environment Study, Cycling in Cities Research Program (2008). Site Observation Form.

[12] Shu X, Wilson N. Use of guard/girder/restraining rails. TCRP Res Results Digest. 2007;82:1–37. http://www.tcrponline.org/PDFDocuments/TCRP_RRD_82.pdf. Accessed 19 Jul 2016.

[13] Wikipedia contributors. Rail profile (2016). Wikipedia, The Free Encyclopedia.

[14] Schepers JP. A safer road environment for cyclists. TU Delft, Delft University of Technology; 2013. http://repository.tudelft.nl/assets/uuid:fe287480-25cc-4b7d-a6d6-1ca2b5976331/Paul_Schepers1.pdf. Accessed 18 Apr 2016.

[15] Cripton PA, Shen H, Brubacher JR (2015). Severity of urban cycling injuries and the relationship with personal, trip, route and crash characteristics: analyses using four severity metrics. BMJ Open.

[16] Ledsham T, Liu G, Watt E, Wittmann K (2013). Mapping Cycling Behaviour in Toronto.

[17] Ontario Ministry of Transportation. Cycling Skills: Ontario’s Guide to Safe Cycling. Undated. http://www.mto.gov.on.ca/english/safety/pdfs/cycling-skills.pdf. Accessed 15 Jan 2016.

[18] Alta Planning and Design (2008). Bicycle Interactions and Streetcars: Lessons Learned and Recommendations.

[19] Wagenbuur M. Junction design in the Netherlands, Bicycle Dutch. https://bicycledutch.wordpress.com/2014/02/23/junction-design-in-the-netherlands/. Accessed24 Jan 2016.

[20] Vision Zero Initiative. http://www.visionzeroinitiative.com Accessed 19 July 2016.

[21] World Health Organization. International classification of diseases: 10th revision. 2016. http://www.who.int/classifications/icd/en/. Accessed 18 Apr 2016.

